# The French Muséum national d’histoire naturelle vascular plant herbarium collection dataset

**DOI:** 10.1038/sdata.2017.16

**Published:** 2017-02-14

**Authors:** Gwenaël Le Bras, Marc Pignal, Marc L. Jeanson, Serge Muller, Cécile Aupic, Benoît Carré, Grégoire Flament, Myriam Gaudeul, Claudia Gonçalves, Vanessa R. Invernón, Florian Jabbour, Elodie Lerat, Porter P. Lowry, Bérangère Offroy, Eva Pérez Pimparé, Odile Poncy, Germinal Rouhan, Thomas Haevermans

**Affiliations:** 1 Direction des collections, Muséum national d’histoire naturelle, CP 39, 57 rue Cuvier, Paris, Cedex 05 75231, France; 2 Sorbonne Université, UPMC Univ Paris 06, MNHN, CNRS, EPHE, Institut de Systématique, Évolution, Biodiversité (ISYEB), CP39, 57 rue Cuvier, Paris, Cedex 05 75231, France; 3 Archéozoologie, archéobotanique UMR 7209 (LaBex BCDiv) Centre National de la Recherche Scientifique—CNRS, Muséum national d’histoire naturelle, CP 39, 57 rue Cuvier, Paris, Cedex 05 75231, France; 4 Missouri Botanical Garden, P O Box 299, St Louis, Missouri 63166, USA

**Keywords:** Plant ecology, Plant evolution, Taxonomy, Biodiversity, Population dynamics

## Abstract

We provide a quantitative description of the French national herbarium vascular plants collection dataset. Held at the *Muséum national d’histoire naturelle*, Paris, it currently comprises records for 5,400,000 specimens, representing 90% of the estimated total of specimens. Ninety nine percent of the specimen entries are linked to one or more images and 16% have field-collecting information available. This major botanical collection represents the results of over three centuries of exploration and study. The sources of the collection are global, with a strong representation for France, including overseas territories, and former French colonies. The compilation of this dataset was made possible through numerous national and international projects, the most important of which was linked to the renovation of the herbarium building. The vascular plant collection is actively expanding today, hence the continuous growth exhibited by the dataset, which can be fully accessed through the GBIF portal or the MNHN database portal (available at: https://science.mnhn.fr/institution/mnhn/collection/p/item/search/form). This dataset is a major source of data for systematics, global plants macroecological studies or conservation assessments.

## Background & Summary

The dataset (Data Citation 1) of the Paris vascular plant herbarium (P) contains information on the collection of angiosperms, gymnosperms and vascular cryptogams held by the *Muséum national d’histoire naturelle* (MNHN). This collection constitutes, together with the non-vascular plant collections (PC), France’s National Herbarium, which houses a total of ca. 8 million specimens. The collection at P includes ca. 6 million specimens mostly mounted on herbarium sheets, along with alcohol-preserved specimens (held in the ‘alcoothèque’), dry fruits and seeds (the ‘carpothèque’), wood samples (the ‘xylothèque’), pollen slides (the ‘palynothèque’), histological slides (the ‘histothèque’) and samples preserved in silica-gel for genetic studies (the ‘silicagelothèque’). The holdings cover all vascular plant orders worldwide, with a particularly rich record of specimens from France, former French colonies, current French overseas departments and territories, as well as countries that were extensively explored by early French botanists such as China and Brazil. The main body of the collection (the ‘general collection’), which is physically arranged according to a systematic classification system, was built through donations, inter-institutional exchange programs and field expeditions conducted by the MNHN and collaborators. It holds an estimated 200,000 type specimens (about 96% of which have been databased) and historically important collections such as those of Jussieu and Lamarck.

The origin of the Paris herbarium dates back to the late 17th century with the constitution of the first systematic herbaria at what was then called the *Jardin royal des plantes médicinales* (Royal Garden of Medicinal Plants), founded 1635. The collection started to resemble the modern herbarium we know today after the French Revolution and the establishment in 1793 of the *Muséum national d’histoire naturelle*. Today’s P collections are thus the inheritance of over 350 years of botanical activity^1^.

Between 2008 and 2012, the Paris herbarium was completely renovated, a process that included a building overhaul and the systematic reconditioning of all specimens to comply with modern curation standards. The entire collection was moved from fixed cabinets to compactors shelves, thus improving its general state of curation. A major databasing initiative was undertaken in conjunction with the renovation and a massive specimen imaging effort was conducted on a scale never before attempted^2^.

To date, the P collection dataset (Data Citation 1) includes ca. 5,400,000 specimens, which covers about 90% of the estimated total size of the collection. Images are available, under a CC-BY licence for 99% of the specimens, 16% of which are accompanied by complete collection label information, along with the plant name and the family in which it is placed (this material is hereafter referred to as ‘fully databased’).

The P herbarium is not only a globally important source of specimens for taxonomic and morpho-anatomical studies, but it has also become a major and highly promising source of data that can inform a wealth of new investigations dealing with systematics, biodiversity conservation, the impacts of climate change, plant evolution and many other subjects.

## Methods

### Constitution of the physical collection

The oldest herbarium specimens deposited at P, which date back to 1558, are contained in the herbarium of Jehan Girault, who studied surgery in Lyon. They were incorporated into the historical herbarium of the Jussieu family but are now stored and curated separately. The first systematic effort to constitute an ‘herbarium’ was, however, that of Joseph Pitton de Tournefort (1656–1708)^3,4^, who initially acquired specimens from France, and particularly from around Paris, to serve as a working base for his *Élements de Botanique*^5^, later translated into Latin under the title ‘*Institutiones rei herbariae’*^6^. Tournefort’s herbarium was subsequently enriched with his own collections from the Middle East and was personally donated by him to King Louis XIV^3,7,8^.

It was during the reign of King Louis XV that interest in botany significantly increased in France. The king’s passion for the sciences, combined with the influence within his Court of scientists such as Guy-Crescent Fagon (1638–1718) and Louis Guillaume Le Monnier (1717–1799), created a thriving climate for botany at the *Jardin royal*. The path initiated by Tournefort was followed by scientists such as Sébastien Vaillant (1669–1722), Antoine de Jussieu (1686–1758), Bernard de Jussieu (1699–1777), Jean Baptiste de Lamarck (1744–1829) and Antoine Laurent de Jussieu (1748–1836), each of whom constituted a personal herbarium while also contributing to the emergence of modern botany. In parallel with the development of these natural history collections (referred to in French as a ‘*cabinet d’histoire naturelle*’), the king funded several expeditions spanning the globe with a declared aim of conducting scientific studies. These included expeditions by Joseph de Jussieu (1704–1779) to South America, André Michaux (1746–1802) to the Middle-East and then to North America, Jean Baptiste Christian Fusée-Aublet (1720–1778) to what is now French Guiana, René Louiche Desfontaines (1750–1833) to the Atlas mountains, Louis Antoine de Bougainville (1729–1811) and Philibert Commerson (1727–1773) around the world, and Jacques-Julien Houtou de La Billardière (1755–1834) to Lebanon^3,9^. Some of the specimens collected during these trips were included in Le Monnier’s private herbarium, which was later acquired by Jules Paul Benjamin Delessert (1773–1847), whose herbarium is now kept in Geneva (G). Duplicates of some of these specimens can also be found in Vaillant herbarium, now housed at P^3^.

During the French Revolution, the *Jardin royal* and its collections were nationalized and in 1793 became the *Muséum national d’histoire naturelle*. Desfontaines, while holding the Chair of Botany at the *Muséum*, organised the National Herbarium (in the modern sense of the word) between 1793 and 1802 (refs 3,10), which comprised a general herbarium based on Vaillant’s collections enriched with Fagon’s herbarium^3^. For historical reasons Desfontaines kept Pitton de Tournefort’s collection separate. At the time the collection included about 25,000 specimens, including both vascular and non-vascular plants now housed in both parts of the National Herbarium (P and PC). By 1808 the collections had outgrown the Muséum’s *Cabinet d’Histoire naturelle* and were transferred to the *Maison Léger*, a building situated close to the *Hôtel de Magny* within the modern day *Jardin des Plantes*, which had been purchased in 1802 but was demolished in 1878 (ref. 1).

During the first half of the 19th century many societies were established for the exchange of herbarium specimens. They persisted for a century and grew into one of the major sources of material for P. In parallel, from the beginning of the 19th century through the middle of the 20th century, material was received form missionaries and ‘correspondents’, botanists based in the field who collected at least in part for the MNHN and sent specimens to Paris. With the start of colonisation in the 1850s, military and colonial administrators also contributed greatly to P. Together these added significantly to the size and richness of today’s herbarium. By 1835, due to the tremendous increase in the size of the collection, it again became necessary to relocate the P herbarium, which was transferred to a newly built ‘Gallery of Botany and Mineralogy’, likewise within the *Jardin des Plantes*^1^.

The beginning of the 20th century was a period of rapid growth for P, with major donations enriching its holdings (Table 1). By 1935, the collections, which contained more than 4 million specimens, were stored in 4 separate buildings and most were inaccessible for research. Thanks to the pugnacity of Paul Henri Lecomte (1856–1934), who held the Chair of Botany starting in 1906, and a donation from the Rockefeller Foundation, the present herbarium building was constructed to host the collections and facilitate their study^1^. The collection was dramatically enriched with material from Madagascar during the period when Jean-Henri Humbert (1887–1967) held the Chair of Botany, from 1931 to 1958, reflecting his strong interest in the island’s flora, which was sustained by his successors, including André Aubréville (1897–1984), who held the Chair from 1958 to 1971 and was also interested of all of the other French-speaking territories (former colonies)^10^.

After World War II and the subsequent period of decolonisation, the rate at which the Paris collection grew started to decrease. Field work conducted by MNHN botanists and exchanges between herbaria became the main sources of new acquisitions. The main activities of botanists at P remained focused on the floras of the former colonies and French overseas territories, of which the holdings at P are particularly strong. The closure of several botanical institutions within France that were unable to maintain collections for financial reasons provided another source of material for P (Table 1).

By the end of the 20th century the collections had outgrown the capacity of the original storage facilities in the 1935 building and the decision was made in 2005 to undertake a complete renovation of the herbarium in order to increase its capacity and improve specimen storage conditions. Starting in 2008 the collections were moved into newly installed compactors, making it possible to integrate the accumulated backlog of some 830,000 previously unmounted specimens and accommodate new specimen acquisition for the next three decades. This operation included a comprehensive resorting of the entire collection into a linear sequence following the APG III classification system^11^. Concomitantly a mass digitisation project was undertaken^1,12,13^, which led to the integration of high resolutions images of all specimens into a dedicated database as described below, accompanied by basic information (accession number, family, genus, specific epithet, geographic region of origin and image) for those that had not already been fully databased.

### Databasing of the physical collection

**Figure 1:**
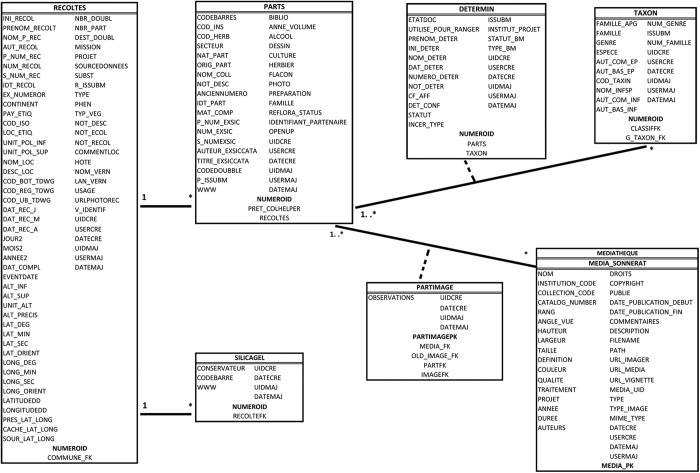
Unified Modeling Language (UML) diagram of the main tables of the Sonnerat database. It presents the column header disposition through the tables, and the tables relations.

The first discussions about biodiversity databases began at P during the mid-1970s and the first tool specifically designed to manage herbarium information was developed during the mid-1980s with the creation of the Vaillant database^14,15^. Commissioned in 1993, the current herbarium database, named Sonnerat (Data Citation 1), was developed to serve as a computerised catalogue of the P collections. It was named after Pierre Sonnerat (1748–1814), a French naturalist, illustrator and explorer who identified, described and illustrated numerous species, mainly from Asia and Mauritius. The database has now developed into the repository for the other herbarium collections of the MNHN (registered in the index herbarorium as PC & PAT^16^) as well as for the network of herbaria in French-speaking countries and the partners in the e-ReColNat project (see below). Sonnerat serves as the core source for data delivered by other portals (e.g., GBIF, e-ReColNat). As with all of the other shared collection databases at the MNHN, Sonnerat is a relational database and was developed under Oracle. The main tables constituting Sonnerat, with the header of the conlumns they contain and the relations between them are presented in a Unified Modeling Language (UML) diagram in Fig. 1. To manage the data and facilitate their dissemination over the web, a specific tool was developed under Java starting in 1997 by the *Centre Informatique du Muséum*. It was initially named JCIM (for Java [solution] of the *Centre Informatique du Muséum*) and later renamed JACIM in order to avoid internal confusion with the name of the Ichtyological collection database (GICIM). Updates and upgrades are performed internally by a dedicated team in the *Direction des Services Informatiques*.

Data are published online through the Data Portal of the MNHN^17^. Additionally a Darwin Core archive (http://rs.tdwg.org/dwc/index.htm) is regularly generated and available on GBIF data portal (Data Citation 1). As for every collection database, the archive is generated using the Occurrence core. All field definitions (Darwin Core Terms) are available on the Darwin Core website (at: http://rs.tdwg.org/dwc/terms/index.htm#occurrenceindex). The archive itself is constitute of 4 text files containing the structural information (the occurrence core, which here comes together with 3 extensions: multimedia, identifier and identification), an eml file containing the data themselves, and an xml file doing the link in between the source database (here Sonnerat) and the archive.

Considering the sheer size of the collection at P, its digitisation has been a tremendous task and indeed this process is still ongoing to deal with the holdings that remain to be scanned (1% of the total; an estimated 10% of the collection remains to be databased).

The first databasing efforts were concentrated on the day to day activities of the herbarium, in particular recording specimens being sent on loan and new accessions related to studies conducted by MNHN researchers. Initially between 20,000 and 30,000 new entries were made per year, but since initiation of the mass digitisation of the collection carried out under the Renobota project starting in 2008, work on the database has changed radically and become a key tool for herbarium management, focusing mainly on updating and enriching the data and in particular adding collection information to those entries that have not yet been fully databased. Moreover, during the Renobota project only a single entry was made per specimen, even for those on which more than one distinct collection has been mounted (this is particularly true for the Cosson herbarium). A Nikon D800 camera mounted on a Kaiser R1 copy stand system is currently being used to image all new specimens and for image updates.

In order to increase the rate of databasing in addition to this routine herbarium work, systematic data entry efforts were developed from 2001 onwards, involving numerous skilled personnel (Supplementary File 1). Continental African Asteraceae, Malagasy Fabaceae-Caesalpinioideae and Papilionoideae, as well as specimens from Mayotte Island and the Union of the Comoros were treated during this process^15,18,19^.

**Figure 2:**
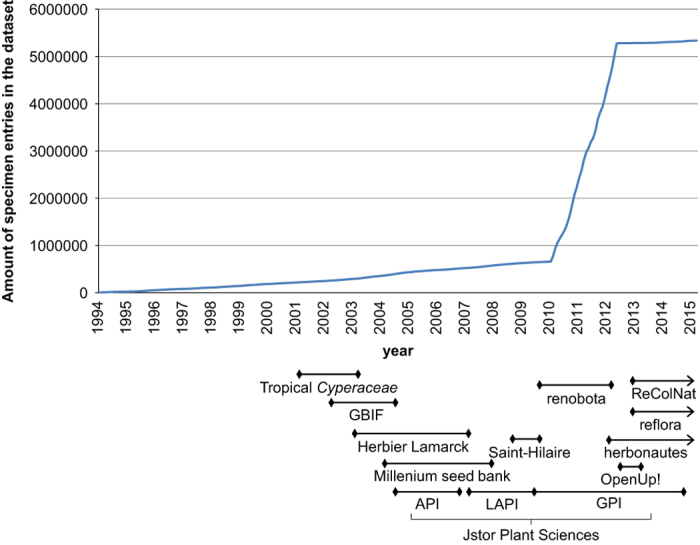
Growth of the P dataset (number of specimen entries) per year according to project.

The substantial dataset that is available today^17^ (Data Citation 1) was also achieved through the MNHN’s active involvement in several national and international databasing projects, each focusing on distinct aspects of data acquisition corresponding to different uses of the rich botanical collection housed at P. As shown in Fig. 2, the most notable of these projects, in terms of the amount of data captured, is the Renobota project, which would not have been possible without input from precursor projects, and for which much work remains to be done in order to achieve full digitisation of the collection. The main projects based at P are presented below and their output is fully integrated in the general dataset (Data Citation 1).

### Cyperaceae from tropical Asia and tropical Americas digitising project (2001–2003)

Funding: Institut Klorane, MNHN

Digitising for the Cyperaceae from tropical Asia and tropical Americas project focused on all Cyperaceae specimens from these regions. Over 8,000 specimens from Asia and 23,000 from America were treated over a three year period.

### Global biodiversity information facility project (2002–2004)

Funding: GBIF France

The GBIF project was the first global databasing effort undertaken at P. It focused on tropical material of two families: over 34,000 specimens of Orchidaceae and 16,800 specimens of Solanaceae where databased, and a search for type specimens was implemented.

### Millennium seed bank project (2004–2008)

Funding: Royal Botanic Gardens, Kew

This project, which focused on databasing specimens from Mali, Burkina Faso and Madagascar, was conducted in collaboration with the Kew Millennium seed bank. A total of 31,000 specimens from 120 families (45 for Madagascar and 75 for Burkina Faso and Mali) were fully databased, including post-facto calculated geolocation data.

### Global plants projects (African plant initiative 2004–2006, latin American plant initiative 2007–2008, global plant initiative 2009–2015)

Funding: Andrew W. Mellon Foundation

These three projects funded by the Mellon Foundation aimed at databasing and producing high-resolution (600 dpi) scans of all the type specimens held in herbaria throughout the world, including P. The first phase, the African Plant Initiative (API), which operated from 2004 to 2006, focused on the African type specimens. It was then followed by the Latin American Plant Initiative (LAPI), from 2007 to 2008, and finally the Global Plant Initiative (GPI) followed from 2009 to 2015, which sought to scan all types from throughout the world, with the exception of Europe.

These three projects resulted in the databasing and digitisation of 185,937 specimens (including 177,045 type specimens and 8,892 specimens of nomenclatural interest), amongst which 45,500 types were located for the first time in the collection, requiring verification. For most of these specimens, the digitisation process was done with Herbscan units, in which a scanner is mounted upside-down, which were provided by the Royal Botanic Gardens, Kew. For thick specimens as well as especially fragile material, high definition Leaf photographic equipment mounted on a motorized bench was used. A 300 dpi copy of each image is linked to the data on the MNHN server (Data Citation 1), while a 600 dpi image, together with the most important data, is duplicated on the Global Plants on JSTOR portal (materials contributed by the Muséum National d’Histoire Naturelle (P) available at: http://plants.jstor.org/partner/P).

### Herbier Lamarck project (2004)

Funding: Centre national de la Recherche scientifique

This project sought to digitise the totality of Jean-Baptiste Lamarck’s herbarium, in its current composition, as part of a project portal dedicated to Lamarck’s scientific legacy. All of the ca. 19,000 specimens were scanned after having been given an accession number, and are available on a dedicated portal (http://www.lamarck.cnrs.fr/herbier.php). When a record for an accession in the Lamarck herbarium also exists in Sonnerat, a link is automatically provided. The data from the Lamarck portal will be fully integrated into the P dataset in the near future (Data Citation 1).

### Auguste de Saint-Hilaire virtual herbarium project (2009)

Funding: Instituto de Botanica de São Paulo(IBt), Centro de Referência em Informação Ambiental (CRIA ), Fundação de Amparo à Pesquisa do Estado de São Paulo (Fapesp), Fundação Vitae, Institut des Herbiers Universitaires, CLF, Clermont-Ferrand

This project, conducted remotely in Brazil, concentrates on digitising the herbarium material collected by Auguste de Saint-Hilaire (1779–1853) in Brazil from 1816 to 1822. Specimen data are presented in a dedicated portal under the form of a virtual herbarium, where the sheets are linked to the relevant pages of Saint-Hilaire’s field notebooks. Data are harvested twice a day from Sonnerat by the virtual herbarium. To date P has contributed over 9,300 specimens, and the rate of data capture has increased significantly since 2012 mainly due to the databasing efforts of the Reflora project^20^ (available at: http://hvsh.cria.org.br/hv).

### Renobota project (2008–2013)

Funding: Muséum national d’histoire naturelle, Paris (MNHN)

Renobota is the abbreviated name used to designate the massive digitisation of the herbarium specimens at P as part of the MNHN’s comprehensive renovation project of the National Herbarium. This integrated project, carried out from 2008 to 2013, involved renovation of both the collection and the building (doubling its storage capacity, offering new working space, and providing controlled environmental condition for specimen storage).

Work on the collections comprised two distinct and complementary operations. The first involved processing the huge backlog of material accumulated over decades, including unmounted and unfiled specimens that had long been unavailable for consultation and research. This material was sorted by the herbarium staff and ca. 120,000 duplicate specimens were dispatched to botanical institutions around the world, with priority given to the countries in which the collections had been made. A private company, Grahal, was contracted during a four year period to carry out this operation, which resulted in the mounting of 830,000 herbarium sheets of vascular plants. The specimens were arranged by family and packed, awaiting their incorporation into the general collection as part of the second operation.

The second step aimed to digitise and rearrange the entire holdings of vascular plants at P. Oce Business Services France was contracted to carry out a multi-objective process at a dedicated site selected for this specific purpose. The objectives included: integration of the newly mounted specimens among the remainder of the collection, reconditioning with new genus folders, mass digitisation of all specimens, rearrangement according to the APG III classification system and, within each family, to a new sequence of generic and of geographic areas. Each sheet received a barcoded accession number label (if one had not previously been given) and was then scanned, with the exception of type specimens (stored in red folders, which had already been treated as part of the Global Plants Science projects; see above) and Vaillant specimens (stored in green folders and treated in part by the GPI team). Essential data were captured (accession number, geographic region and taxon name), which is the basic information necessary to link the virtual collection (i.e., the scanned images) to the physical collection (the specimens). The resulting data and associated images were then uploaded into the Sonnerat database^12,13^. Over 5,000,000 specimens were digitised and for more than 90% of them, a new database entry was created in Sonnerat. The project resulted in a massive number of specimen entries, each associated with an image, but only limited collection label data were captured (Data Citation 1).

As a result of the Renobota project, the operating procedures at P as well as access for the botanical community to the herbarium’s facilities and collections have been significantly improved, although managing the process of updating both the virtual and real herbaria remains a considerable challenge.

### Les Herbonautes project (2012-present)

Funding: MNHN, Fondation de la Maison de la Chimie, e-ReColNAt (ANR-11-INBS-0004), Muséum national d’histoire naturelle, Paris (MNHN)

**Figure 3:**
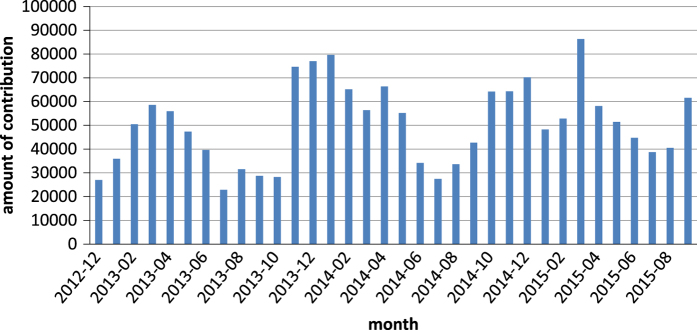
Monthly contributions to *Les Herbonautes* from its launch (December 2012) to the end of September 2015.

*Les Herbonautes* (http://lesherbonautes.mnhn.fr/) is an ongoing participatory science project aimed at transcribing the information written on herbarium labels through a dedicated web-based portal. Images are shown to registered web volunteers through thematic projects (called ‘missions’) designed by researchers curating collections and focusing on scientific research needs. The web volunteers (called ‘herbonauts’) transcribe, for each image, data on the collection locality, date, collector, collection number, identification, and geolocation, as requested. The data quality is cross-checked: in order to be validated, the same data must be captured independently by several different contributors. Tests of data quality have shown comparable levels between data obtained using this method and professional standards. Since the opening of the website in December 2012, 37 missions have been launched, 35 of which have been completed. As of September 9, 2015, a total of 2,181 contributors have examined 119,956 specimens, providing 1,685,738 entries (i.e., responses to a single question). After three years, the public’s enthusiasm for databasing herbarium specimens remains high, although a certain seasonality can be seen: more contributions are made in winter than summer (Fig. 3).

The initial version of the *Les Herbonautes* portal was developed as part of the Renobota project and a Version 2, developed through the e-ReColNat project, was released in January 2016. This new version allows for better follow up by both the project managers and the public. The data generated through *Les Herbonautes* can now be transferred to the Sonnerat database (Data Citation 1) through the ReColNat portal.

### Open Up! project (2013)

Funding: Europeana grant for OpenUp!

The OpenUp! project aims to provide images from natural history museum collections to the Europeana portal (http://www.europeana.eu/). The images, selected based on criteria for potential interest to the broad public, were chosen from among those generated during the Renobota project. In parallel, curation of the entire set of the taxonomic data in the Sonnerat database was performed (Data Citation 1). Data from 385,286 specimens of P were provided to the Europeana portal (available at: http://www.europeana.eu/).

### Reflora project (2013–2016)

Funding: Conseil National pour la Recherche (CNPq)-Vale

The Reflora project aims to create a portal giving access to data from every botanical collection relating to the Brazilian flora. All of the ca. 300,000 images produced by the Renobota project from specimens collected in Brazil are being sent to the Herbarium Collection of the Rio de Janeiro Botanical Garden, where teams of botanists and students are databasing them. Data are then sent back to P for incorporation into Sonnerat (Data Citation 1). To date, some 130,000 images have been provided to Reflora and data derived from 30,000 of these have been added to the P dataset. The global data resulting from the Reflora project are available on a dedicated virtual herbarium (http://reflora.jbrj.gov.br/reflora/herbarioVirtual/).

As part of this project, and in partnership with the National Museum of the Federal University of Rio de Janeiro, a virtual herbarium was established that is dedicated to the specimens collected by Auguste Glaziou (http://glaziou.cria.org.br), build on the model of the Auguste de Saint-Hilaire virtual herbarium.

### e-ReColNat project (2013–2019)

Funding: PIA Agence Nationale de la Recherche-11-INBS-0004, Muséum national d’histoire naturelle.

This project aims to facilitate online access to the biodiversity collections held in France. At P, it consists mainly of a quality control monitoring of the Sonnerat database, while also developing new accessibility tools. A platform (*Explore*, available at: https://explore.recolnat.org/), bringing together resources from most of the French natural history collections and allowing an advanced browsing and data enrichment process, is under development. *Explore* will allow enrichment of the databased specimens through participatory science projects, directly through the platform and through a dedicated version of *Les Herbonautes*.

Additionally, using the structure created by e-ReColNat, a backlog of ca. 70,000 specimens from P have been digitised by Picturae at Heiloo^21^, after having been mounted. These mainly include new material received recently, loans returned too late to be included in the Renobota project, and some material left over from the earlier mounting initiative.

### Additional databasing

Most of the databasing efforts described above concerned the general herbarium collection at P. The standing-alone collections housed at MNHN (referred to as ‘historical collections’) were not part of the Renobota digitisation process. However, some were treated in part during various projects, as follows:

- The Humboldt & Bonpland Herbarium was fully digitised and databased by the GPI team. 3525 entries are available in the P dataset.
- The Lamarck herbarium was completely digitised during a dedicated project. Data remain to be uploaded to Sonnerat. To date, 4053 specimens are available in the P dataset.
- The American specimens of the Jussieu herbarium, estimated to represent ca. 10% of the material in this collection, are being digitised and databased as part of the Reflora project; 13,500 specimens (including the American material and some other specimens) have so far been databased.
- Various specimens from other stand-alone collections were databased as part of the regular specimen loan procedure. The most significant of these are 543 specimens from the herbarium of Léon Mercurin (1898–1994), 230 from the Michaux herbarium, 133 from the Tournefort herbarium, 19 from Desfontaines *Flora Atlantica* herbarium, 18 from the herbarium of Elias Magloire Durand (1794–1873), and 6 from the Adanson herbarium.

Data published through GBIF: http://collections.mnhn.fr/ipt/resource.do?r=mnhn-p (Data Citation 1)

### Sonnerat database taxonomic coverage

During the Renobota project, the organisation of the collections at P was modified to follow the linear family sequence based on the APG III classification^11^. However, the placement of some specimens could not be updated at that time, and for various reasons, some collections are still filed under the same family as prior to the renovation, which followed the index developed by Théophile Durand (1855–1912) based on Bentham & Hooker’s classification^22^.

**Figure 4:**
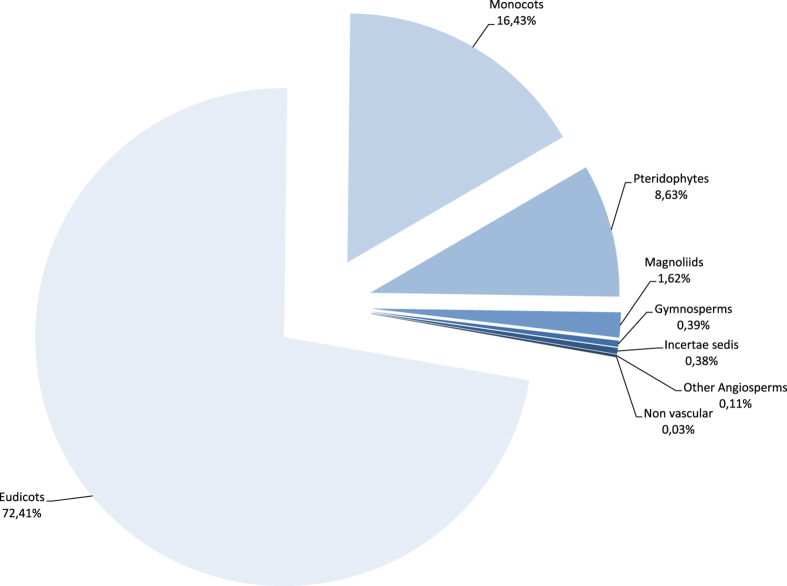
Taxonomic coverage of the P dataset in term of specimens. Following the APG IV classification for Angiosperms^33^ (entries through 24/03/2016).

The P herbarium includes all vascular plant orders. A recent comparison (as of 19 October 2015) with The Plant List v.1.1 (http://www.theplantlist.org/) showed that 34% of the accepted species names are represented by one or more specimens, and an analysis of the non-matching data indicated that this was due to errors both in the P dataset and The Plant List. Once these issues have been resolved and remaining material at P is databased, coverage is estimated to be between 33 and 40% of all vascular plant species. As depicted in Fig. 4, the eudicots constitute the largest portion of the collection, with ca. 72%, followed by the monocots (ca. 17%) and the pteridophytes (ca. 9%).

### P dataset spatial coverage

**Figure 5:**
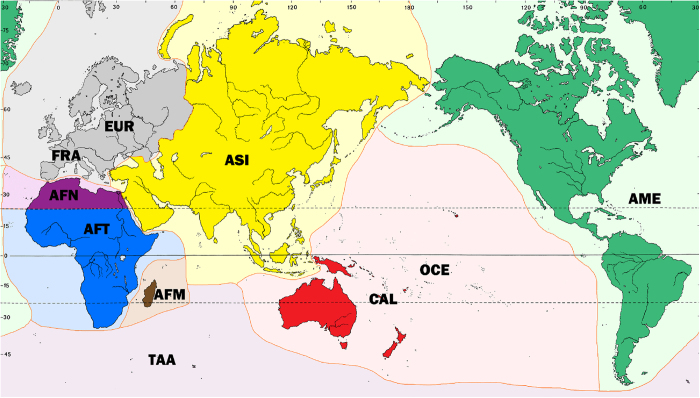
Breakdown by geographic sectors at P.

The collections at P, which are global in scope, have been divided into 12 sectors based on place of collection to facilitate curation, access and regional studies (Fig. 5). The distribution of the accessions among these sectors is heterogeneous (Table 2). The FRA sector, corresponding to metropolitan France, is one of the largest, with ca. 970,000 specimens. The two continents with the largest number of specimens, Europe and Africa, have each been sub-divided into distinct sectors. For Europe, this dates back to the donation of de Candolle’s collection^8^ and to the creation of the France herbarium, which has evolved into the FRA sector, separate from the EUR sector, which encompasses the rest of Europe. The European flora comprises the largest portion of the P herbarium, with 29% of the holdings, a situation shared with other natural history collections at the MNHN^23^. Somewhat later, Africa was also sub-divided based on biogeographic considerations into AFT (Tropical Africa), AFM (Madagascar and the Western Indian Ocean islands) and AFN (Northern Africa), which together represent ca. 25% of the collections at P. The taxonomic composition within each sector is indicated in Table 2.

To date, 16% of the specimens included in Sonnerat are fully databased, resulting from a wide range of efforts (see detailed projects above). The percentages of specimens from which data are fully captured differ from one geographic sector to another (Table 2). All of the remaining specimens processed during the Renobota project are databased at least for family, genus and species names (when available), along with main geographical sector, but not for information related to the individual specimen (collector name, collection number, date, locality, etc.).

Europe, France and Northern Africa have a very low percentage of fully databased specimens. Similarly, the very low rate for cultivated material reflects the scientific focus of P botanists, which is primarily on wild plants. By the time the Reflora project is completed, data capture for specimens from Brazil should increase the rate for America significantly. Oceania is divided into two sub-sectors: CAL for New Caledonia and OCE for the rest of the region. The P herbarium contains at least 200,000 specimens from New Caledonia, but a technical issue makes it impossible to calculate the exact number, so the following numbers are provided for Oceania as a whole. The level of databasing for this sector is comparatively high since P botanists are working on the Polynesian^24,25^ and New Caledonian floras, which account for most of the material available. The collections from Madagascar are among the most studied and actively curated at P, and have been since the early 20th century. As a result they are the most extensively databased (full data have been captured for 35%). Special efforts have been made on the collections from the circum-austral islands, which are relatively small in number (85% have been fully databased). Finally, the stand-alone (historical) collections were not treated as part of the Renobota imaging process so the only records are those resulting from projects that fully captured the associated data. As a consequence, the very low proportion for which data entry is not complete (0.02%) corresponds to background error rates in databasing.

**Figure 6:**
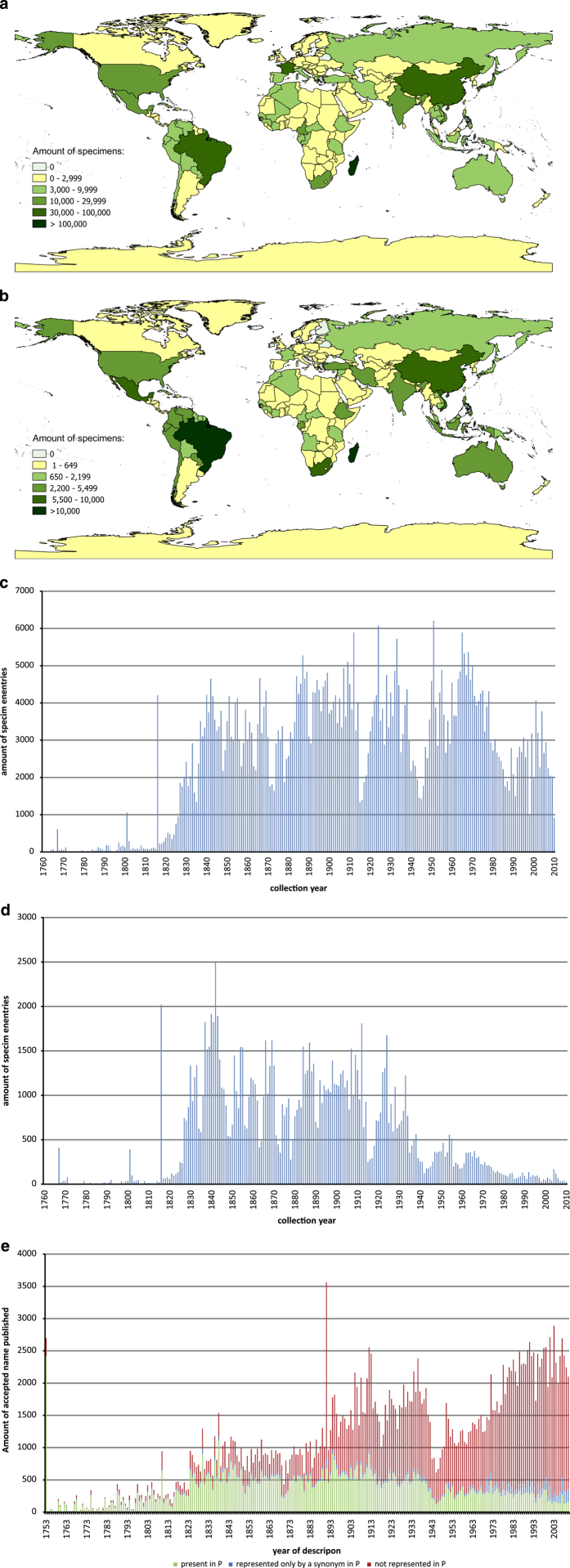
Geographical and chronological composition of P dataset. (**a**). Geographical origin of vascular plant specimen in P (based on the 759,568 specimens bearing a valid country code (ISO 3166)) (**b**). Geographical origin of nomenclatural type specimens (based on the 186,809 bearing a valid country code (ISO 3166)) (**c**). Chronological distribution of vascular plant specimens in P by year of collection. (based on the 631,017 specimens with collection date between 1760 and 2010) (**d**). Chronological distribution of nomenclatural type specimens of vascular plants in P by year of collection. (based on the 130,189 type specimens with collection date between 1760 and 2010) (**e**). Number of accepted names of vascular plants by year of description (source: The Plant List^34^) represented as such (green), just by a synonym (blue), or not represented at all (red) in the P dataset (entries up to 04.04.2016).

Because of the heterogeneity in the proportion of fully databased specimens among geographic sectors, the distribution by country shown in Fig. 6a, which takes into consideration only these records, very likely does not reflect the true situation for the entire collection at P. For example, European and Northern African countries are under-represented while Madagascar and Brazil are over-represented. This map nevertheless provides important information on the geographical provenance of the specimens in the P herbarium. To facilitate interpreting this figure, we have calculated a factor of representativeness for each sector by dividing the average rate of full data capture per sector by the overall average for the collection as a whole:

- Within Europe, metropolitan France is under-represented, as indicated by a factor of 0.52, and the rest of Europe by a factor of 0.38. However, excluding metropolitan France, the Mediterranean flora is better covered at P than northern Europe.
- Within Africa, Madagascar is over represented by a factor 2.22, tropical continental Africa is close to the overall average with a factor 1.03, and northern Africa is significantly under represented, with a factor 0.37. Specimens from the former French colonies are well represented, but it is notable that South Africa, Ethiopia and Tanzania are as well.
- For the Americas, the factor is 1.12, indicating that this sector is over-represented among the records for which data have been fully captured. While there is no way to calculate the factor for Brazil, it will surely exceed the value for the sector as a whole given that several projects are focusing on its flora.
- For Asia, the collections are slightly over-represented, with a factor of 1.09. The value for former Indochina is expected to be the highest, whereas those for India, Japan and Siberia (most of the databased specimens from Russian at P were collected in Siberia) as well as China are expected to be lower than the average for the sector. The expected over-representation for Indochina reflects the historical relationship with this region and the flora project coordinated at P.
- For Oceania, the factor of 1.50 indicates an over-representation, most of which reflects the impacts of major flora projects for French Polynesia and New Caledonia, areas from which the collections at P are particularly rich. P is also noteworthy for its rich folding of material from Australia.
- For the circum-autral territories, the collections are over represented, as indicated by a factor of 5.54, although the small sample size makes it difficult to interpret this figure.

The P herbarium is very rich in type specimens from throughout the world, a large proportion of which were systematically databased during the three Global Plants Projects. The distribution of types by country of collection (Fig. 6b) largely confirms the general trend observed for the collection as a whole. However, given that no systematic searches were made to detect previously unrecognised types in the African sector during the API project (only types already placed in distinctive red folders were considered), a significant but unknown number of types remains to be found among the continental African collections. Because none of projects supported by the Mellon Foundation concerned the European flora, collections from this sector are underrepresented in this map. Additionally, even though the GPI team conducted a systematic search for type specimens from America, Asia and Oceania, the Reflora project has shown that a systematic review of the collections from a given country by specialists will surely reveal additional, previously unrecognised types.

By comparing the country of collection (as indicated by the country isocode in the fully databased portion of the herbarium) with the broad geographical sector assigned to the entire collection, an estimate can be made of the geographical error rate, including mismatches, invalid country codes, and unassigned sector codes (even though in principle each specimen must be assigned to a sector). However, because it is sometimes difficult to determine the country from which a specimen was collected, especially for older material, empty country codes were not considered as errors. We obtained a correct match for over 97% of the records.

### Temporal coverage (year 1558-present)

The oldest plants preserved at P are sheets made out of the flower garlands found on the mummy of Ramesses II, which were sent for study to the MNHN^4^. These few extremely old specimens are, however, exceptions, as are those held in Jehan Girault’s collection, which dates from 1558. The number of specimens in the collection began to increase significantly during the late 17th century, but it is often impossible to determine the precise date of collection for this oldest material as this information was almost never mentioned by collectors on specimens made before the 19th century.

The number of specimens at P according to collection date (Fig. 6c) shows an increase starting in the 1820s. This is in large part related to the activities of French missionaries and later to the exploration that preceded colonisation and formation of botanical societies, which sent duplicates to the MNHN and those who held other collections that are now kept at P. The large number of specimens recorded for 1816 is an artefact of the fact that Auguste de Saint-Hilaire’s specimens, which were collected between 1816 and 1821, were all entered as 1816 because no precise date is generally indicated.

Three major wars impacted the growth of the collections at P during the last two centuries:

- the Franco-Prussian War (1870), followed by the French Commune (1871)
- the First World War (1914–1918)
- the Second World War (1939–1945)

War is not particularly compatible with botany, although botanical activities never totally ceased during times of conflict^26^. The decrease in the number of collections added per year at P at the end of the 1960s parallels documented decreases at other historical natural history collections in Australia, Germany and France^23,27,28^. In Paris this can be linked to decolonisation, but also to a decrease of funding for activities that generate material deposited in herbaria and to a societal perception of such collections as unimportant or unnecessary^23,27,28^. The apparent renewal of interest since the 1990s, as suggested by the increase in growth at P, might be linked with the development of the database Sonnerat and the systematic databasing of new collections made by MNHN botanists, facilitating the production of specimen labels, which could this explain the apparent higher rate of collection during these years. The lower rate of databasing of specimens in the last decade is seen among many natural history collections database^23^ and might be linked to the fact that significant numbers of specimens still being studied have not yet been incorporated into herbaria.

Figure 6d shows the same information specifically for type specimens up to the end of World War II, after which a drop in the proportion of types in the collection can be seen, even though the worldwide number of accepted taxa described per year has increased. As depicted in Fig. 6e, a high portion of the taxa described in the first century after Linnaeus’ work are represented at P, and this proportion has declined ever since.. Moreover, over the last five to six decades, a growing number of species are filed in the Paris herbarium under a synonym rather than the currently accepted name, which in large part reflects the increasing amount of curatorial effort needed to ensure that the collection is regularly updated as taxonomic changes are made.

### Databased samples collection and preservation methods

Vascular plants collection (P) of the *Muséum national d’histoire naturelle* (MNHN—Paris), active databasing period: 1993–2015 (Data Citation 1).

#### Specimen preservation methods

The collection consists in dry specimens mounted on herbarium sheets, dry wood samples, dry fruits and seed, samples preserved in alcohol, tissues and pollen mounted on microscope slides, tissue samples preserved in silica-gel.

It is mostly composed of herbarium sheets, which are divided into a general systematic collection (5.7 million specimens, 95% databased), ordered according to the APG III classification for the Angiosperms, and into several stand-alone collections of historical importance (estimated at 150,000 specimens, 14% databased), which are curated separately. In addition to the herbarium, P also includes some complementary collections:

- Histology (70,000 slides, not yet databased, 20,000 of which came from the collection of Philippe van Tieghem)
- Palynology (45,000 slides, not yet databased, ca. 12,000 of which have vouchers in the P herbarium)
- Specimens preserved in alcohol (estimated to comprise 15,000 specimens, 4% databased)
- Wood collection (estimated at 14,000 specimens, 83% databased). An significative curation effort, including data management, is currently being made on this particular collection
- Dry fruit and seed collection (estimated to include 28,000 specimens, 6% databased)
- Samples preserved in silica-gel for molecular studies (ca. 7,300 samples, 100% databased)

#### Sampling protocol

No single sampling protocol can be distinguished, the acquisition of the collection tremendously varies depending on the institute from which the sample is derived or the collector. An ideal label for those specimens consists in: taxonomic information, collection information (who?; where?, when?) completed by GPS coordinates and projection system, descriptive or ecological data which cannot physically be preserved on the specimen, ethnobotanical information (vernacular names, uses). Moreover, for most of the older, historical specimens, no information is available on the sampling methodology used.

### Use of the dataset

The P dataset (Data Citation 1) is widely used for a great range of botanical or organisms interactions researches encompassing systematics, evolution, phylogenetic diversity, conservation biology, taxonomy as well as monographs and flora treatments^18,19,24,25,29–32^.

## Data Records

The ‘Darwin Core Archive Vascular plants collection (P)’ of the Muséum national d’histoire naturelle (MNHN—Paris) (Data Citation 1) is a Darwin Core Archive (version 1.0) file, encoded in UTF-8 and permanently available for download at the following address (http://collections.mnhn.fr/ipt/resource.do?r=mnhn-p), as well as on the GBIF depository (Data Citation 1).

## Technical Validation

The data quality of the dataset has been thoroughly checked with several taxonomic authorities such as The Plant List (http://www.theplantlist.org/), Tropicos (http://www.tropicos.org.) and IPNI (The International Plant Names Index, available at: http://www.ipni.org/). The quality of data entered through the various small scale projects cited in the method section consists in matching of the physical label with the database entry for each record and has been carefully human-checked. Database administrators cleaned the database following the massive Renobota digitising process and varying orthography in authors or locality names have been normalized creating authorities lists. An analysis of the non-matching data for scientific names indicated that this was due to errors both in the P dataset and The Plant List which were subsequently addressed. The data contributed by the Herbonautes Project also benefits from a high redundancy (the same label is randomly given to several officers and the data is considered correct when a consensus is reached between them).

## Usage Notes

The dataset is fully Darwin Core format compliant and can be downloaded as a whole through the GBIF portal (Data Citation 1) or following application of specific filters (taxonomy, geography, collector, types).

## Additional Information

**How to cite this article:** Le Bras, G. *et al.* The French Muséum national d’histoire naturelle vascular plant herbarium collection dataset. *Sci. Data* 4:170016 doi: 10.1038/sdata.2017.16 (2017).

**Publisher’s note:** Springer Nature remains neutral with regard to jurisdictional claims in published maps and institutional affiliations.

## Supplementary Material



Supplementary Information

## Figures and Tables

**Table 1:** Major acquisitions at P.

**Year**	**Botanist(s)/Institution**	**Details**
1805	Aimé Bonpland (1773–1858) and Alexander von Humboldt (1769–1859)	Donation of an important part (ca. 3 500 specimens) of the herbarium constituted during their trip in South and central America. It is kept separate as a stand-alone collection^3,35^.
1822	Augustin Pyrame de Candolle (1778–1841)	Donation of specimens used for his work over the *Flore Françoise*. It led to the creation of a section dedicated to the Flora of France^8,36^.
1833	René Louiche Desfontaines (1750–1833)	Donation of the specimens used for his *Flora Atlantica*^37^, curated as a stand-alone collection. The remaining Desfontaines herbarium is now kept in Florence (FI).
1857	Jussieu family	Donation of the private herbarium of the Jussieu’s family. The collection is curated as a one of the most important stand-alone collection at P^7,8^.
1872	Adrien de Jussieu (1797–1853)	Donation of a collection of 5.300 specimens from France, that were integrated to the general collection^7^.
1886	Jean Baptiste de Lamarck (1744–1829)	Purchase of the Lamarck Herbarium from the University of Rostock. It is estimated to count ca. 19,000 specimens and is stored among the major standing-alone collections^38–40^.
1904	Emmanuel Drake del Castillo (1855–1904)	Donation of a very rich collection counting ca. 500,000 specimens. Include several herbaria, such as Franchet’s, de Franqueville’s (itself including the herbaria of Achille Richard (1794–1852) and his father, and of Ernst Gottlieb Steudel (1754–1821)), Vesian’s, Lenormand’s or Schultz’s. An exhaustive catalogue has been established by Bureau in 1904^8,39,41^.
1904	Ernest Saint-Charles Cosson (1819–1889)	Donation of a very rich collection counting ca. 500,000 specimens. It is constituted by Cosson’s original collections, but as well herbaria such as Moquin-Tandon’s, Bunge’s, Fée’s and Schultz-Bipontinus’. He was a specialist for the Mediterranean flora^8,39,42,43^.
1906	Jean Baptiste Louis Pierre (1833–1905)	Donation of a herbarium which is an important collection for the former Indochinese territories^8^.
1907	Auguste François Marie Glaziou (1833–1906)	Donation of an important herbarium for Brazilian flora^39^.
1924	Michel Adanson (1727–1806)	Purchase of 24 095 specimens collected by the botanist during his travels to Senegal and in France. It is kept as a separate stand-alone collection.
1925	Roland Napoléon Bonaparte (1858–1924)	Donation of an important collection of ca. 2,000 cardboard box of Pteridophytes, which includes part of the original collections of Konrad Christ and Christian Luerssen^1^.
1954	Jean Baptiste Christian Fusée-Aublet (1720–1778)	Purchase of part of the collection constituted by the botanist in French Guiana. The collection has been purchased in 1778 by Jean-Jacques Rousseau, a few weeks before his death^44^. It is kept as a stand-alone collection.
1970	Faculty of Pharmacy of Paris	Donation of ca. 50,000 specimens.
1974	Caen University	Donation of the ca. 270,000 specimens, containing amongst other the original collections from Jules Dumont d’Urville (1790–1842).
1978	Faculty of Pharmacy of Paris	Second donation of ca. 40,000 specimens.

**Table 2:** Number of specimens per continent and geographic sector, and taxonomic coverage for each continent.

**Continent**	**Sector**	**Specimens per sector**	**Percentage of specimens with complete collection data capture**	**Specimens per continent**	**Taxonomic composition**
**EUROPE**	FRA	966,793	7.95%	1,573,830	Eudicots: 75.80%Monocots: 17.93%Pteridophytes: 5.67%Magnoliids: 0.13%Gymnosperms: 0.30%Incertae sedis: 0.09%Other angiosperms: 0.08%
	EUR	607,037	5.88%		
**AFRICA**	AFT	703,767	15.95%	1,351,437	Eudicots: 73.54%Monocots: 17.82%Pteridophytes: 6.23%Magnoliids: 1.75%Gymnosperms: 0.17%Incertae sedis: 0.42%Other Angiosperms: 0.08%
	AFM	411,967	34.20%		
	AFN	235,703	5.64%		
**AMERICA**	AME	1,067,320	17.29%	1,067,320	Eudicots: 68.78%Monocots: 15.79%Pteridophytes: 12.28%Magnoliids: 2.34%Gymnosperms: 0.32%Incertae sedis: 0.39%Other Angiosperms: 0.10%
**ASIA**	ASI	933,888	16.89%	933,888	Eudicots: 71.53%Monocots: 13.98%Pteridophytes: 10.36%Magnoliids: 2.90%Gymnosperms: 0.55%Incertae sedis: 0.47%Other Angiosperms: 0.20%
**OCEANIA**	OCE & CAL	351,791 [including at least 200,000 for CAL]	23.07%	351,791	Eudicots: 66.31%Monocots: 13.54%Pteridophytes: 16.30%Magnoliids: 2.37%Gymnosperms: 1.02%Incertae sedis: 0.31%Other Angiosperms: 0.16%
**CIRCUM AUSTRAL**	TAA	3,003	85.40%	3,003	Eudicots: 42.22%Monocots: 33.70%Pteridophytes: 23.91%Gymnosperms: 0.13%Incertae sedis: 0.03%
**CULTIVATED**	CLT	64,352	1.54%	64,352	Eudicots: 89.53%Monocots: 9.38%Pteridophytes: 0.43%Magnoliids: 0.15%Gymnosperms: 0.23%Incertae sedis: 0.26%Other Angiosperms: 0.02%
**HISTORICAL COLLECTION**	HIS	22,191	99.98%	22,191	Eudicots: 61.94%Monocots: 20.72%Non vascular: 8.39%Pteridophytes: 6.64%Magnoliids: 2.08%Gymnosperms: 0.09%Incertae sedis: 0.02%Other Angiosperms: 0.13%
Based on the 5,367,812 specimens bearing sector information—99.59% of the collection (as of 24 March, 2016).					
