# Supplementary material for: The French Muséum national d’histoire naturelle vascular plant herbarium collection dataset
Source: Sci Data. 2017 Feb 14;4:170016. doi: 10.1038/sdata.2017.16 (PMC5308200; doi:10.1038/sdata.2017.16)
Supplement: Supplementary Information [file sdata201716-s2.doc]

**Supplementary File 1 : Contributors to P dataset constitution per project mentioned in the article**

**Cyperaceae from tropical Asia and tropical Americas digitising Project (2001-2003)**

Hélène Falaise (data provider), Jean-Noël Labat (project manager)

**Global Biodiversity Information Facility Project (2002-2004)**

Isabelle Bouchart (data provider), Benoît Carré (data provider), Séverine Dramet (data provider), Véronique Durand (data provider), Hélène Falaise (data provider), Thomas Haevermans (data provider), Bérangère Offroy (data provider), Marc Pignal (project manager), Céline Pisivin (data provider), Christophe Reveillard (data provider)

**Millennium Seed Bank Project (2004-2008)**

Benoit Carré (data provider), Vincent Bourguignon (data provider), Mehdi Jabbori (data provider), Della Hopkins (Kew - project Manager), Jean-Noël Labat (project manager), Audrey Lestel (data provider)

**Global Plants Projects (African Plant Initiative 2004-2006, Latin American Plant Initiative 2007-2008, Global Plant Initiative 2009-2015)**

Amandine Allard (API, LAPI, GPI - data provider), Karine Augiron (API - data provider), Ehoarn Bidault (GPI - data provider), Marie Bouissière (LAPI, GPI - data provider, project manager), Vincent Bourguignon (LAPI, GPI - data provider), Benoît Carré (LAPI, GPI - data provider), Fanny Chabas (GPI - data provider), Juliette Chatard (LAPI, GPI - data provider), Pascale Chesselet (LAPI, GPI - project manager), Nicolas Cottin (API - data provider), Vanessa Damianthe (LAPI, GPI - data provider), Véronique Darricau-Suhone (API, LAPI - data provider), Marion Depraetère (GPI - data provider), Véronique Durand (API - data provider), Olivier Durbin (LAPI, GPI - data provider), Xavier Filereau (API - data provider), Grégoire Flament (API, LAPI - data provider), Eva Fryde-Azoulay (GPI - data provider), Gabriel Gagnier (GPI - data provider), Rémi Girault (API - data provider), Mehdi Jabbori (LAPI - data provider), Lionel Kervran (API - data provider), Jean-Noël Labat (API, LAPI, GPI - project manager), Charlotte Labbe (GPI - data provider), Gwenaël Le Bras (LAPI, GPI - data provider, data manager), Elodie Lerat (LAPI, GPI - data provider), Caroline Loup (GPI - project manager), Lora Martin (API - data provider), Marion Martinez (GPI - data provider), Thomas Millerand (API - data provider), Vinciane Mossion (GPI - data provider), Charles Munoz Nantes (GPI - data provider), Sylviane Murat (LAPI, GPI - data provider), Bérangère Offroy (API, LAPI - data provider), Marc Pignal (API - project manager), Edwinstaël Ramanantsoa (LAPI, GPI - data provider), Marie-Laurent Randrihasipara (API, LAPI, GPI - data provider, data manager, project manager), Jules Souquet-Basiège (LAPI - data provider), Paule Terres (GPI - data provider), Jérémy Tritz (GPI - data provider), Frédéric Tronchet (LAPI - data provider)

**Herbier Lamarck Project (2004)**

Vincent Leguy (data provider), Yuria Mizuta (data provider), Delphine Usal (data provider), Pietro Corsi (project manager), Marc Pignal (project manager), Stéphane Pouyllau (data manager), Xavier Aubriot (data manager)

**Auguste de Saint-Hilaire virtual herbarium Project (2009)**

Marc Pignal (data provider, project manager)

**Renobota Project (2008-2013)**

Cécile Aupic (collection curator, collection identifier, data provider), Gérard Aymonin (collection identifier), Simon Chagnoux (data manager), Sabine Comtet-Andriamanjatoarivo (collection manager, data provider), Thierry Deroin (collection curator, collection identifier, data provider), Bérenger Dulac (data manager), Hélène Falaise (collection manager, data provider), Grégoire Flament (collection manager, data provider), Jacques Florence (collection curator, collection identifier, data provider), Myriam Gaudeul (collection curator, collection identifier, data provider), Claudia Gonçalves (data provider), Jean-Christophe Grouard (collection manager, data provider), Thomas Haevermans (collection curator, collection identifier, data provider), Sovanmoly Hul (collection curator, collection identifier, data provider), Caroline Loup (collection manager, data provider), Porter Lowry (collection curator, collection identifier, data provider), Bérangère Offroy (collection manager, data provider), Eva Pérez Pimparé (data provider), Peter Phillipson (collection curator, collection identifier, data provider), Marc Pignal (collection curator, collection identifier, data provider, data manager), Odile Poncy (collection curator, collection identifier, data provider, project manager), France Rakotondrainibe (collection identifier, data provider), Christophe Reveillard (collection manager, data provider), Germinal Rouhan (collection curator, collection identifier, data provider), Corinne Sarthou (collection curator, collection identifier, data provider), Grahal Company (collection provider), OCE Company (data provider)

**Les Herbonautes Project (2012-present)**

Simon Chagnoux (data manager), Marc Pignal (project manager), Germinal Rouhan (“mission” manager), Bruno Dennetière (“mission” manager), Benoît Carré (“mission” manager), Gwenaël Le Bras (“mission” manager), Marc L. Jeanson (“mission” manager)

**Open Up! Project (2013)**

Gwenaël Le Bras (data provider, data manager)

**Reflora Project (2013-2016)**

Nathalie Allain (data provider), Amandine Allard (data provider), Fanny Chabas (data provider), Fabrice Clermont (data provider), Bérenger Dulac (data manager), Claudia Gonçalves (data provider, project manager), Vanessa R. Invernón (data provider), Elodie Lerat (data provider), Marc Pignal (project manager)

**e-ReColNat Project (2013-2019)**

Bérenger Dulac (data manager), Michel Guiraud (project manager), Julien Husson (data manager), Gwenaël Le Bras (data manager), Eva Pérez Pimparé (project manager), Marc Pignal (project manager)
